# Multiplex pyrosequencing assay using AdvISER-MH-PYRO algorithm: a case for rapid and cost-effective genotyping analysis of prostate cancer risk-associated SNPs

**DOI:** 10.1186/s12881-015-0186-x

**Published:** 2015-06-25

**Authors:** Jérôme Ambroise, Valentina Butoescu, Annie Robert, Bertrand Tombal, Jean-Luc Gala

**Affiliations:** Center for Applied Molecular Technologies (CTMA), Institut de Recherche Expérimentale et Clinique (IREC), Université catholique de Louvain, Clos chapelle-aux-champs B1.30.24, Brussels, 1200 Belgium; Service d’Urologie, Institut de Recherche Expérimentale et Clinique (IREC), Cliniques Universitaires Saint-Luc, Université catholique de Louvain, Brussels, 1200 Belgium; Epidemiology and Biostatistics Department (EPID), Institut de Recherche Expérimentale et Clinique (IREC), Université catholique de Louvain, Brussels, 1200 Belgium

**Keywords:** Multiplex, Pyrosequencing, Polymporphisms, Prostate

## Abstract

**Background:**

Single Nucleotide Polymorphisms (SNPs) identified in Genome Wide Association Studies (GWAS) have generally moderate association with related complex diseases. Accordingly, Multilocus Genetic Risk Scores (MGRSs) have been computed in previous studies in order to assess the cumulative association of multiple SNPs. When several SNPs have to be genotyped for each patient, using successive uniplex pyrosequencing reactions increases analytical reagent expenses and Turnaround Time (TAT). While a set of several pyrosequencing primers could theoretically be used to analyze multiplex amplicons, this would generate overlapping primer-specific pyro-signals that are visually uninterpretable.

**Methods:**

In the current study, two multiplex assays were developed consisting of a quadruplex (n=4) and a quintuplex (n=5) polymerase chain reaction (PCR) each followed by multiplex pyrosequencing analysis. The aim was to reliably but rapidly genotype a set of prostate cancer-related SNPs (n=9). The nucleotide dispensation order was selected using SENATOR software. Multiplex pyro-signals were analyzed using the new AdvISER-MH-PYRO software based on a sparse representation of the signal. Using uniplex assays as gold standard, the concordance between multiplex and uniplex assays was assessed on DNA extracted from patient blood samples (n = 10).

**Results:**

All genotypes (n=90) generated with the quadruplex and the quintuplex pyroquencing assays were perfectly (100 %) concordant with uniplex pyrosequencing. Using multiplex genotyping approach for analyzing a set of 90 patients allowed reducing TAT by approximately 75 % (*i.e.*, from 2025 to 470 min) while reducing reagent consumption and cost by approximately 70 % (*i.e.*, from ∼229 US$ /patient to ∼64 US$ /patient).

**Conclusions:**

This combination of quadruplex and quintuplex pyrosequencing and PCR assays enabled to reduce the amount of DNA required for multi-SNP analysis, and to lower the global TAT and costs of SNP genotyping while providing results as reliable as uniplex analysis. Using this combined multiplex approach also substantially reduced the production of waste material. These genotyping assays appear therefore to be biologically, economically and ecologically highly relevant, being worth to be integrated in genetic-based predictive strategies for better selecting patients at risk for prostate cancer. In addition, the same approach could now equally be transposed to other clinical/research applications relying on the computation of MGRS based on multi-SNP genotyping.

## Background

Since 2005, a large number of Genome-Wide Association Studies (GWAS) have identified Single Nucleotide Polymorphisms (SNPs) associated with more than 300 complex diseases and traits [1]. For prostate cancer (PCa), 47 risk-associated SNPs have been identified [2] that are essentially found in 8q24 and 17q chromosomal regions although not strictly restricted to these areas. Most of these SNPs have been tested in large population surveys as stand-alone predictors.

Considering that each SNP has only a moderate association with PCa, Multilocus Genetic Risk Scores (MGRSs) were computed in different studies to assess the cumulative association of multiple SNPs. Zheng et al. assessed the cumulative effect of five selected SNPs [3]. Compared to men without any of these risk genotypes, the odds ratio for PCa was 4.47 (2.93-6.80) in those having four of them or more. Kader et al. computed MGRS based on 33 established PCa risk-associated SNPs and demonstrated the potential added value of the score for PCa risk prediction [4]. More recently, a MGRS computed from a set of nine published SNPs (rs1016343, rs16901979, rs6983267, rs4242382, rs10993994, rs10896449, rs4430796, rs1859962, and rs5945619) improved the performance of a clinical risk-calculator in predicting prostate biopsy result [5]. The predictive performance of the integrated clinico-genetic model (AUC = 0.781) was higher than the predictive performance of the clinical score alone (AUC = 0.770). This set of nine risk-associated SNPs was selected from the breast and prostate cancer cohort consortium (BPC3) results [6], according to reported allelic odds ratio, prevalence and potential linkage disequilibrium.

While approaches for identifying men at high risk of PCa are still needed, the costs of genetic testing are currently too high to perform large-scale screening [7]. Consolidating a broader use of genetic testing in PCa early detection algorithms will require a cheaper and faster though equally high throughput and reliable procedure. Pyrosequencing is a cost-effective DNA sequencing technique that has many applications, including rapid SNP genotyping. The chemiluminescent signal produced during the reaction is detected in the pyrosequencer and displayed in a pyro-signal (*i.e.*, a pyrogram™) which is then translated into the corresponding nucleotide sequence. Usually, genotyping a single SNP is carried out with one separate reaction in one well (*i.e.*, a uniplex experiment). Genotyping of multiple SNPs using uniplex pyrosequencing requires therefore performing multiple simultaneous or successive reactions for each patient, which impacts reagent costs and Turnaround Time (TAT). A more efficient alternative would be to use simultaneously a set of several pyrosequencing primers in a single well (*i.e.*, a multiplex reaction).

Duplex (n=2) and triplex (n=3) pyrosequencing applications for SNP genotyping were recently developed using LBP [8], CTLA-4 [9], or CYP2C19 [10] target genes. In these applications, nucleotide dispensation orders were carefully selected and successfully avoided overlapping primer-specific signals. However, only one gene with two to three different SNPs were assayed. For some multiplex pyrosequencing applications, overlapping primer-specific signals are unavoidably created and visually uninterpretable. Accordingly, the AdvISER-M-PYRO software was recently and specifically developed to allow the analysis of overlapping pyro-signals generated from multiplex reactions (i.e, the comprehensive set of each peak height characterizing a well-defined pyro-sequence in uniplex experiments or a combination thereof in multiplex experiments) [11, 12]. In parallel, the pyrosequencing nucleotide dispensation order was improved by developing the SENATOR (“SElecting the Nucleotide dispensATion Order”) algorithm. AdvISER-M-PYRO is based on the modelling of multiplex pyro-signals as a sparse representation of elements (named atoms) from a standardized learning dictionary that includes corresponding uniplex pyro-signals. The first application of SENATOR and AdvISER-M-PYRO consisted in genotyping alterations underlying bacterial resistance to *β*-lactam antibiotics [12].

In the present study, two multiplex assays were developed consisting of a quadruplex and a quintuplex PCR, each followed by pyrosequencing analysis for genotyping a set of PCa-related SNPs (n=9) [5]. The dispensation order was selected using SENATOR and multiplex pyro-signals were analyzed with an adapted version of AdvISER-M-PYRO (named AdvISER-MH-PYRO), which integrates a new function allowing bi-allelic SNP genotyping. The analytical reagent costs, waste production and TAT of conventional uniplex and new multiplex pyrosequencing assays were compared.

## Methods

### Patients and DNA extraction

Both multiplex pyrosequencing assays were developed and validated on DNA extracted from patient blood (n = 10). These patients had been included in a previous study [5] and provided informed consent, following approval of the study by the Ethics Committee (Comité d’Ethique Hospitalo-Facultaire, Cliniques Universitaires Saint-Luc - Université Catholique de Louvain, Brussels). The extraction of genomic DNA was performed from peripheral blood lymphocytes using the EZ1 DNA Blood kit and BioRobot EZ1 (Qiagen, Leusden, The Netherlands), according to the manufacturer’s protocol.

### Selection of the pyrosequencing dispensation order

A list of all unique nucleotide sequence (UNS) expected to be found in the 9 selected genomic regions was compiled (Table 1). Considering that pyrosequencing experiments were designed with reverse primers for some SNPs (Tables 1 and 2), the complementary sense sequence was computed for the corresponding UNS. Allocation of each of the nine SNPs either to quadruplex or quintuplex assay was made according to optimal PCR conditions commonly used for individual SNP genotyping. Then, the SENATOR [12] function was used to generate a suitable nucleotide dispensation order for both quadruplex and the quintuplex assays.

**Table 1:** List of selected SNPs (n=9) and their corresponding MAF, OR and UNS

SNP	Chromosome	Allelic	MAF ^*a*^	Unique Nucleotide	Forward	Quadruplex(n=4)
		OR ^*a*^		Sequences (UNS)	Reverse	Quintuplex (n=5)
rs1016343	8	1.25	0.21	TTCCCTCCCA	Reverse	4
				TTCCCTCTCA		
rs10993994	10	1.23	0.47	TGACGTCGAA	Forward	4
				TGATGTCGAA		
rs16901979	8	1.44	0.18	ATCTGGCAAA	Forward	4
				CTCTGGCAAA		
rs5945619	X	1.23	0.26	ACTCCCGCCG	Reverse	4
				ACTCCCGCTG		
rs10896449	11	0.84	0.4	GCTGAAAATT	Reverse	5
				GCTGAAAGTT		
rs1859962	17	1.19	0.39	TGATGAACAC	Forward	5
				GGATGAACAC		
rs4242382	8	1.4	0.16	CCACAGGCCC	Forward	5
				CCGCAGGCCC		
rs4430796	17	0.8	0.46	GATGCTGCAT	Forward	5
				AATGCTGCAT		
rs6983267	8	0.81	0.43	TGAAAGGCAC	Reverse	5
				TGAAAGTCAC		

^*a*^OR: Odds Ratio, MAF = Minor Allele Frequency

**Table 2:** PCR and pyrosequencing primers and primer concentrations for each selected SNP before and after concentration adjustment

SNP	Quadruplex (n=4)	Multiplex PCR			Multiplex PYRO	
	Quintuplex (n=5)		Conc.(*μ*M)			
		Primer	Initial ^*a*^	Adjusted ^*a*^	Primer	Conc.(*μ*M)
rs1016343	4	F: 5’-Biot-TCAGGGCAATTACGGAATAACA-3’	0.1	0.1	5’-TGAAGCTGTGAGTAATCA-3’	0.4
		R: 5’-AATCTAAAGAATGGGGGTCAGAG-3’	0.1	0.1		
rs10993994	4	F: 5’-CTCTCCTCCTCTGCTCTTTTAGGT-3’	0.1	0.1	5’-TTGTTATCATTCCCAA-3’	0.4
		R: 5’- Biot- AGGCAAAGCTGCATCAAACT-3’	0.1	0.1		
rs16901979	4	F: 5’-GTGGGGTCTTTGTTGTGGA-3’	0.1	0.1	5’-AATGATTTAGCATTACTTAT-3’	0.4
		R: 5’-Biot-TTATGTTCAGAGCGGTTGAATG-3’	0.1	0.1		
rs5945619	4	F: 5’-Biot-CAGGAAGGGGAACATCACACT-3’	0.1	0.05	5’-CTTGCGGGAGTCTCA-3’	0.4
		R: 5’- ACGGCTACTATGAGATGAGGAAAC-3’	0.1	0.05		
rs10896449	5	F: 5’-Biot-GGGCCACAGGGAACAA-3’	0.1	0.15	5’-TGACATTCCCCTTCTTA-3’	0.4
		R: 5’-GCCTTTTCAATGAACCCACA-3’	0.1	0.15		
rs1859962	5	F: 5’-AATAAGAGGCTGCAGACTTTTCC-3’	0.1	0.05	5’-AAATCCCTGCCCGTG-3’	0.4
		R: 5’-Biot-TAGGCATTCCAAAGATGAAGACTC-3’	0.1	0.05		
rs4242382	5	F: 5’-AAAAGAGGTAACCCAGGGAACA-3’	0.1	0.075	5’-TTGTCCCTCTAGTTATCTTC-3’	0.4
		R: 5’-Biot-GCATAGAGGGACGCTGTCAA-3’	0.1	0.075		
rs4430796	5	F: 5’-ACGTCCCTTCCTCAGCATCTT-3’	0.1	0.3	5’-GGCAGCACAGACTGGA-3’	0.4
		R: 5’-Biot-TGTTCCTGACATGAAGCAACTCT-3’	0.1	0.3		
rs6983267	5	F: 5’-Biot-TCTTCCTATCTCAGCTCCCTATCC-3’	0.1	0.1	5’-AATTCTTTGTACTTTTCTCA-3’	0.4
		R: 5’-GTTGGCTGGCACTGTCTGT-3’	0.1	0.1		

^a^For each SNP, the concentrations of PCR primers were adjusted in order to select the best conditions for balancing the respective contribution of each SNP in multiplex pyro-signals

### Uniplex and multiplex PCR and pyrosequencing

Identification of the nine SNPs was first carried out for all patients (n=10) using uniplex (n=9) PCRs, followed by uniplex pyrosequencing reactions (n=9). All genotypes (n=90) resulting from uniplex pyro-signals were used as gold standard.

In the following steps, quadruplex PCR followed by quadruplex pyrosequencing was carried out on four SNPs, while quintuplex PCR followed by quintuplex pyrosequencing was carried out on the remaining five SNPs (Table 1). Although protocols for individual SNP genotyping differed initially in terms of MgCl2 concentration (2.25 or 3.0 mM) and number of PCR cycles (35 or 40), subsequent condition testing allowed to standardize the PCR multiplex protocol.

Multiplex PCR was carried out in a 50 *μ*L reaction mixture containing 7 or 10 *μ*L (for quadruplex and quintuplex assays, respectively) of the extracted DNA (50 ng), 5 *μ*L of a PCR buffer (100 mM Tris hydrochloride, and 500 mM potassium chloride, pH 8.3), 3mM MgCl2, 1U AmpliTaq Gold®; DNA Polymerase (AmpliTaq Gold®; DNA Polymerase kit from Applied Biosystems®;, Austin, USA), 200 *μ*M of each deoxynucleotide triphosphate (dNTPs: dATP, dCTP, dGTP, dTTP Li-salts from Roche Diagnostics GmbH, Mannheim, Germany) and forward and reverse PCR primers (Table 1) (Eurogentec, Liège, Belgium). Amplification was performed in a 2720 Thermal Cycler (Applied Biosystems®;) using the following conditions: 95 °C for 5 minutes, followed by 40 cycles with denaturation at 95 °C for 40 seconds, annealing at 60 °C for 40 seconds, and extension at 72 °C for 80 seconds, with a final extension step at 72 °C for 7 minutes. Electrophoresis of PCR products was performed on 2 % agarosis gel. Pyrosequencing was then carried out with a pyrosequencer PyroMark PSQ 96 MA Sequencer from Qiagen (Hilden, Germany) on PCR products, using a mixture of the pyrosequencing primers (0.4 *μ*M each) (Table 2), enzymes and substrate (PyroMark Gold®; Q96 Reagents kit, Qiagen) according to the manufacturer’s protocol. Each PCR and pyrosequencing reaction included a negative control. All uniplex and multiplex pyrosequencing reactions were carried out with the selected dispensation orders. They were also compared in terms of analytical reagent costs, production of waste material and TAT.

### Multiplex pyro-signal processing using AdvISER-MH-PYRO

All multiplex pyro-signals were converted into their corresponding genotypes in three successive steps, as described below. Firstly, two standardized learning dictionaries were created, one for the quadruplex, the other for the quintuplex assay. Each dictionary includes a uniplex theoretical pyro-signal for each genotype expected to be found within each genomic region. Aside of the theoretical uniplex pyro-signals (*i.e.*, 7 and 10 in the quadruplex and quintuplex assay, respectively), those generated by uniplex pyrosequencing were also included in both dictionaries. These experimental uniplex pyro-signals were standardized by dividing all peak heights by the corresponding first unitary peak height (FUPH), as previously recommended [12].

**Fig. 1:**
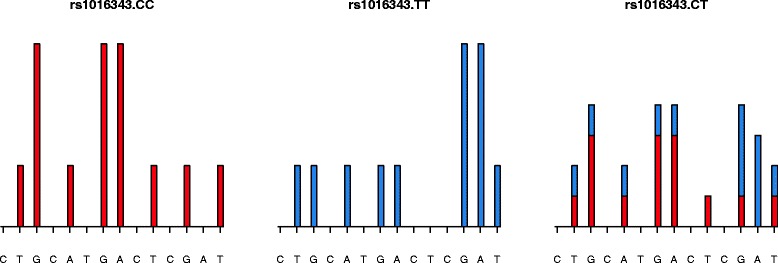
pyro-signals generated by each rs1016343 homozygous (CC and TT) and heterozygous (CT) variants

In a second step, each multiplex pyro-signal was analyzed with AdvISER-MH-PYRO software. While not included with the previous AdvISER-M-PYRO version which was dedicated to bacterial DNA genotyping, a new feature was implemented into AdvISER-MH-PYRO in order to carry out bi-allelic SNP genotyping. For these SNPs, the pyro-signal generated by a heterozygous variant results from the superposition of pyro-signals generated by both corresponding homozygous allelic variants which, as expected, disclose peak heights twice higher than their heterozygous counterparts (Fig. 1). When the contribution of each homozygous and heterozygous variant is computed for a bi-allelic SNP, a correction factor is then applied by the AdvISER-MH-PYRO version, taking this effect into account. AdvISER-MH-PYRO was implemented in an R package (www.uclouvain.be/ctma.html) that can be applied to analyze multiplex signals generated in a broad range of human SNP genotyping applications.

In a final step, the optimal primer concentration was adjusted for each SNP of both multiplex assays. Accordingly, multiplex PCR and pyrosequencing reactions were first carried out with an initial PCR primer concentration (0.1 *μ*M for each SNP) on a first subset of patients (n=3), and contributions of each SNP to the global multiplex pyro-signal were computed with AdvISER-MH-PYRO. Concentrations of PCR primers were then adjusted in order to select the best conditions for balancing the respective contribution of each SNP in multiplex pyro-signals (Table 2).

### Interoperability of the dictionary

Considering that the multiplex pyrosequencing approach, as described in this study, requires building a standardized dictionary based on experimental uniplex pyro-signals, an essential feature of the method appears therefore the interoperability of this dictionary for analyzing multiplex pyro-signals generated by different pyrosequencers. Accordingly, multiplex (quadruplex and quintuplex) pyro-signals generated with the PyroMark Q96 ID sequencer, Qiagen (Hilden, Germany), were analyzed using the same dictionary and AdviSER-PYRO software and compared to original results generated by PyroMark Q96MA.

## Results and discussion

### Selection of the nucleotide dispensation order

SENATOR was used to select a dispensation allowing to differentiate between all UNSs of interest for the current application (Table 1). Nucleotide dispensation orders with 14 (CTGCATGACTCGAT) and 15 (AGATCGCTACGACTG) nucleotides were selected for the quadruplex and quintuplex assays, respectively. They generated theoretical uniplex pyro-signals with low pairwise correlation coefficients, avoiding collinearity between signals which are contained in the dictionary and used as predictors in the penalized regression models within the AdvISER-MH-PYRO function.

For both multiplex assays, the theoretical multiplex signals corresponding to all possible combinations of genotypes were generated. The maximum values of the pairwise correlation coefficients between all multiplex signals were equal to 0.9891 and 0.9892 for the quadruplex and quintuplex assays, respectively. Therefore, the selected dispensation orders proved to generate a specific and unique multiplex pyro-signal for each genotypic combination.

### Uniplex pyrosequencing

Uniplex pyro-signals of all DNA samples (n=10) were obtained with the nucleotide dispensation order corresponding to the quadruplex or quintuplex SNP allocation, and used as gold standard genotype (Table 3).

**Table 3:** Individual genotype (n=10) for selected SNPs (n=9)

Patient	rs1016343	rs10993994	rs16901979	rs5945619	rs10896449	rs1859962	rs4242382	rs4430796	rs6983267
1	C/C	T/T	C/C	T	G/G	G/G	G/G	A/A	G/T
2	C/C	C/C	C/C	T	A/G	T/T	G/G	A/A	G/T
3	C/C	C/C	C/C	C	A/G	T/T	G/G	G/G	G/T
4	C/C	C/C	C/C	T	A/G	T/G	G/G	A/A	G/G
5	C/C	C/T	C/C	T	G/G	T/T	G/G	G/A	T/T
6	T/T	C/T	C/C	T	A/G	T/T	G/G	G/A	T/T
7	C/C	C/T	C/C	T	A/G	T/G	G/G	G/A	G/G
8	C/T	C/T	A/A	T	A/A	T/T	G/G	A/A	G/T
9	C/C	C/C	C/C	C	G/G	T/T	A/G	G/A	T/T
10	C/C	C/C	C/C	T	A/G	G/G	G/G	G/A	G/T

### Adjustment of PCR primer concentration

Regarding the quadruplex pyro-signals before primer adjustment, the average relative signal contribution of rs5945619 was higher (35.3 %) than the three other SNPs (15.9 %, 27.4 % and 21.4 % for rs1016343, rs10993994 and rs16901979, respectively). The primer concentration for rs5945619 was therefore decreased (Table 2).

Regarding the quintuplex pyro-signals before primer adjustment, the average relative signal contribution of rs4430796 (5.8 %) and of rs10896449 (10.1 %) were smaller than rs6983267 (21.6 %), while the average relative signal contributions of the two remaining SNPs were significantly higher (35.6 % for rs1859962 and 25.9 % for rs4242382). The primer concentration was therefore increased for rs4430796 and rs10896449 and decreased for rs1859962 and rs4242382 (Table 2).

### Multiplex PCR and multiplex pyrosequencing

**Fig. 2:**
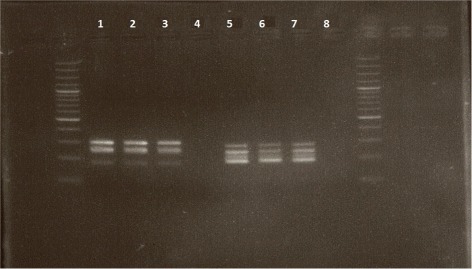
Picture of electrophoresis gel of amplification products obtained with 3 DNA samples and 1 negative control, for the quintuplex (samples 1-4) and quadruplex (samples 5-8) PCR, respectively

Multiplex pyrosequencing was carried out on PCR products produced with adjusted PCR primer concentrations. Gel electrophoresis of the quadruplex and quintuplex PCR products is displayed in Fig. 2. Given the similar sizes of the amplification products for the five analyzed SNPs in the quintuplex PCR (rs6983267 : 295 bp, rs1859962 : 177 bp, rs10896449 : 274 bp, rs4430796 : 254 bp, rs4242382 : 250 bp), only 3 bands were present on the gel (samples 1-4). Regarding the quadruplex PCR (samples 5-8), the picture of the electrophoresis gel shows 4 bands, corresponding to the amplification products for rs1016343 (279 bp), rs10993994 (250 bp), rs5945619 (234 bp) and rs16901979 (191 bp), respectively.

All pyro-signals were analyzed using AdvISER-MH-PYRO. For the quadruplex assay, the average relative signal contribution of rs5945619 (17.2 %) decreased and approached the respective contribution of the other three SNPs (18.1 % for rs1016343, 16.9 % for rs10993994, and 47.7 % for rs16901979) (Table 4).

**Table 4:** Quadruplex (n=4) pyro-signals (n=10): results of AdvISER-MH-PYRO after adjustement of primer concentrations

Patient	rs1016343		rs10993994		rs16901979		rs5945619		R ^*a*^
	Genotype	Contribution ^*a*^ (%)	Genotype	Contribution ^*a*^ (%)	Genotype	Contribution ^*a*^ (%)	Genototype	Contribution ^*a*^ (%)	
1	C/C	4.36 (18.6)	T/T	2.87 (12.2)	C/C	11.35 (48.4)	T	4.85 (20.7)	0.999
2	C/C	5.52 (20.5)	C/C	7.05 (26.2)	C/C	10.71 (39.8)	T	3.66 (13.6)	0.998
3	C/C	4.58 (19.6)	C/C	3.66 (15.6)	C/C	11.09 (47.4)	C	4.09 (17.5)	0.999
4	C/C	2.85 (16.4)	C/C	2.23 (12.8)	C/C	9.78 (56.1)	T	2.57 (14.7)	0.999
5	C/C	4.97 (18.8)	C/T	5.34 (20.2)	C/C	10.63 (40.3)	T	5.44 (20.6)	0.999
6	T/T	3.92 (16.3)	C/T	4.93 (20.5)	C/C	10.2 (42.5)	T	4.95 (20.6)	>0.999
7	C/C	3.98 (17.9)	C/T	3.87 (17.4)	C/C	10.23 (45.9)	T	4.2 (18.9)	0.997
8	C/T	4.87 (18.3)	C/T	5.19 (19.5)	A/A	12.41 (46.6)	T	4.18 (15.7)	0.999
9	C/C	3.05 (18.4)	C/C	1.82 (11.0)	C/C	9.18 (55.3)	C	2.56 (15.4)	0.999
10	C/C	2.72 (16.2)	C/C	2.35 (14.0)	C/C	9.29 (55.2)	T	2.46 (14.6)	>0.999
Average		18.1		16.9		47.7		17.2	

^*a*^For each patient, the genotype and its absolute and relative contributions to the global signal are computed by AdvISER-MH-PYRO for each SNP, R: confidence index traducing the correlation between the observed multiplex pyro-signal and the sparse regression model constructed by AdvISER-MH-PYRO

For the quintuplex assay, the average contribution of rs4430796 (12.6 %) and rs10896449 (19.3 %) were increased while the contribution of rs1859962 (16.6 %) was significantly decreased and approached the contributions of the remaining SNPs (24.4 % for rs6983267 and 26.7 % for rs4242382) (Table 5).

**Table 5:** Quintuplex (n=5) pyro-signals (n=10): results of AdvISER-MH-PYRO after adjustement of primer concentrations

Patient	rs10896449		rs1859962		rs4242382		rs4430796		rs6983267		R
	Genotype	Contriution ^*a*^ (%)	Genotype	Contribution ^*a*^ (%)	Genotype	Contribution ^*a*^ (%)	Genotype	Contribution ^*a*^ (%)	Genotype	Contribution ^*a*^ (%)	
1	G/G	5.51 (19.4)	G/G	8.79 (30.9)	G/G	5.75 (20.2)	A/A	3.24 (11.4)	G/T	5.13 (18.1)	0.998
2	A/G	5.82 (23.2)	T/T	4.05 (16.1)	G/G	6.91 (27.5)	A/A	2.33 (9.3)	G/T	6.00 (23.9)	0.998
3	A/G	5.67 (21.0)	T/T	3.96 (14.7)	G/G	7.63 (28.3)	G/G	3.31 (12.3)	G/T	6.40 (23.7)	0.999
4	A/G	5.07 (20.6)	T/G	3.50 (14.2)	G/G	6.44 (26.1)	A/A	3.30 (13.4)	G/G	6.36 (25.8)	0.996
5	G/G	5.55 (19.3)	T/T	3.52 (12.2)	G/G	7.74 (26.9)	G/A	3.14 (10.9)	T/T	8.86 (30.8)	0.999
6	A/G	7.28 (22.4)	T/T	4.91 (15.1)	G/G	7.47 (23.0)	G/A	5.21 (16.0)	T/T	7.64 (23.5)	0.998
7	A/G	6.35 (21.1)	T/G	4.28 (14.2)	G/G	8.22 (27.3)	G/A	4.28 (14.2)	G/G	7.00 (23.2)	0.996
8	A/A	0.94 (10.7)	T/T	1.47 (16.7)	G/G	2.82 (32.1)	A/A	1.57 (17.9)	G/T	1.99 (22.6)	0.998
9	G/G	4.73 (18.6)	T/T	4.43 (17.4)	A/G	6.80 (26.8)	G/A	2.17 (8.5)	T/T	7.27 (28.6)	0.997
10	A/G	4.23 (14.5)	G/G	3.07 (14.5)	G/G	6.15 (29.1)	G/A	2.50 (11.8)	G/T	5.16 (24.4)	0.999
Average		(19.3)		(16.6)		(26.7)		(12.6)		(24.4)	

^*a*^For each patient, the genotype and its absolute and relative contributions to the global signal are computed by AdvISER-MH-PYRO for each SNP, R: confidence index traducing the correlation between the observed multiplex pyro-signal and the sparse regression model constructed by AdvISER-MH-PYRO

Although the contribution of all SNPs was not perfectly balanced in both assays, all genotypes (n=90) generated with the quadruplex (Table 4) and quintuplex (Table 5) assays were perfectly (100 %) concordant with uniplex pyrosequencing results (Table 3). Moreover, all results were associated with a high confidence index (r >0.997). Accordingly, PCR primer concentrations were not further adjusted.

### Illustration of AdvISER-MH-PYRO

**Fig. 3:**
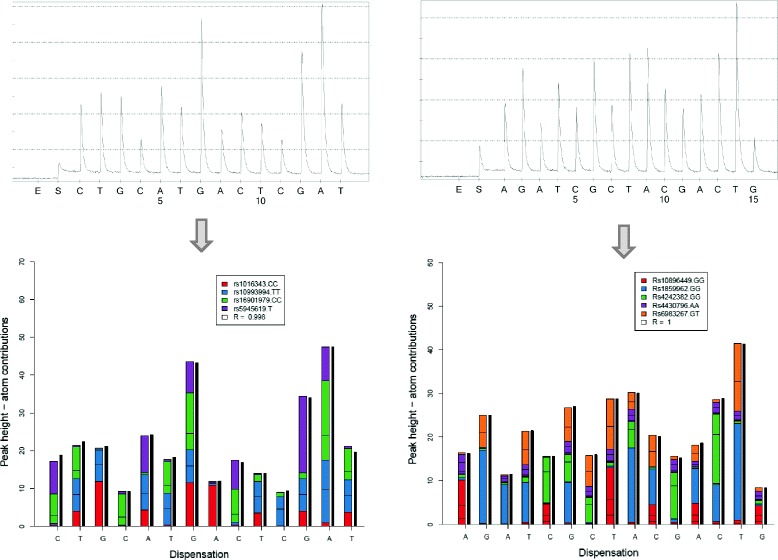
Example of a quadruplex (left) and quintuplex (right) pyro-signal identification with AdvISER-MH-PYRO.The multiplex pyro-signal generated by the pyrosequencing machine is displayed before (top) and after (bottom) the analysis. In both cases, it is represented by vertical black lines. After analysis, the contribution of each atom (*i.e.*, each uniplex pyro-signal within the dictionary) is represented by colored boxes stacked on top of the other

Figure 3 illustrates the results obtained with AdvISER-MH-PYRO when applied on quadruplex (left) and quintuplex (right) pyro-signals generated for the first patient. Both signals are correctly converted into four and five genotypes, respectively.

In both cases, the high confidence index (R=0.998 for quadruplex, R >0.999 for quintuplex) traduces the quasi perfect correlation between the observed multiplex pyro-signal (vertical black lines) and the sparse regression model (colored boxes). As illustrated in Fig. 3, colored boxes, which result from software analysis, fit quasi perfectly the vertical black lines representing the global multiplex pyro-signal produced by the pyrosequencer.

### Interoperability of the dictionary

Irrespective of the equipment used to carry out the pyrosequencing analysis, all genotypes generated with the quadruplex and quintuplex assays were perfectly (100 %) concordant with gold standard results as reported in Table 3.

### Impact on analytical reagent costs, production of waste material and TAT

TAT of uniplex and multiplex methods were also compared. Considering that each PCR and pyrosequencing plates includes 96 wells and that negative controls are required, the comparison was performed on a hypothetical set of 90 patients. Whereas buffy coat, DNA extraction and quantification (30, 30 and 20 min, respectively) were identical for both methods, the estimated TAT of PCR (i.e, 165 min) and pyrosequencing (*i.e.*, 60-70 min) carried out in 9 uniplex versus 2 multiplex analyses, was 2025 ([165 + 60]*9) versus 470 ([165 +70]*2) min, respectively.

Analytical costs for analyzing 9 SNPs with the classical uniplex and multiplex pyrosequencing approach were ∼229 and ∼64 US$/patient, respectively (Table 6).

**Table 6:** Analytical reagent costs (US $)

	Uniplex (n=9)	Quadruplex (n=1)
		+Quintuplex (n=1)
DNA extraction	∼16.5	∼16.5
PCR	∼71.0	∼16.0
Pyrosequencing	∼141.5	∼31.5
TOTAL	**∼229** ***.*** **0**	∼64.0

Using the multiplex approach resulted in an important reduction of waste material produced (∼75 % less than the uniplex method), in terms of quantities of PCR and pyrosequencing plates, pipette tips and reagent bottles (Table 7).

**Table 7:** Waste material produced for a full 96-well plate

	Uniplex (n=9)	Quadruplex (n=1)
		+ Quintuplex (n=1)
PCR plates (n)	9	2
Pipette tips - PCR (n)	936	222
Pyrosequencing plates (n)	9	2
Pipette tips - pyrosequencing (n)	918	211
Bottles pyrosequencing reagents (enzyme+substrate) (n)	18	4

## Conclusions

The present proof-of-concept study aimed to demonstrate the feasibility of SNP multiplex pyrosequencing with the new AdvISER-MH-PYRO algorithm and to assess its impact in terms of cost, TAT and waste material. In this respect, a combination of two multiplex pyrosequencing assays was developed to test in two runs a set of nine prostate cancer-related SNPs. Appropriate nucleotide dispensation orders were selected with the SENATOR function which considers all UNS expected to be found within each genomic region of interest in order to produce uncorrelated uniplex pyrosequencing signals. Multiplex pyro-signals were then analyzed with a new algorithm developed by our team (AdvISER-MH-PYRO). All quadruplex and quintuplex pyro-signals were converted into 4 and 5 genotypes, respectively.

To the best of our knowledge, it is the first time that quadruplex and quintuplex pyro-signals from amplicons generated by a multiplex PCR amplification in a single well are translated into their respective single counterpart and that bi-allelic variants of each target gene are simultaneously identified and assigned. All multiplex results were perfectly (100 %) concordant with uniplex results. The latter were taken as gold-standard in this study, considering that uniplex pyrosequencing distinguishes reliably the specific pattern associated with each of the various genotypes, providing therefore accurate typing results [13], as confirmed in previous studies comparing pyrosequencing versus Sanger sequencing [14, 15]. The analytical reagent costs, waste production and TAT of conventional uniplex and new multiplex pyrosequencing assays were compared. The new multiplex approach allowed to lower costs and waste material production by ∼70 % and ∼75 %, respectively. In an era where research laboratories strive to be more environmentally-friendly, this new multiplex method could therefore contribute to minimize waste disposal and footprint. The comparison of TAT was also clearly in favor of the multiplex approach. TAT was indeed reduced by ∼75 %, without any compromise on results quality and reliability and despite a multiplex pyrosequencing run being 10 min longer due to a higher number of dispensed nucleotides.

While uniplex SNP pyrosequencing does not compete with with faster and more cost-effective methods (TaqMan [16] or Sequenom [17]), current improvements enabling multiplexing makes it a suitable alternative to the latter methods in terms of TAT, cost and waste production. Although not assessed in this study, in-del could also be genotyped in a multiplex experiment with the AdvISER-MH-PYRO algorithm.

Considering that the AdvISER-MH-PYRO software and both dictionaries are available in the corresponding package (www.uclouvain.be/ctma.html), and that PCR conditions have already been optimized, these new assays can also easily be implemented in other laboratories now. As evidenced by this study, multiplexing has proved to be particularly relevant when developing new rapid, robust, reliable, cleaner and cost-effective SNP genotyping assays. While the added clinical value of MGRS based on 9 PCa risk-associated SNPs proved indeed not bring a major benefit as previously discussed [5], it was technologically interesting to repeat this SNP analysis using the multiplex pyrosequencing method, especially because this method is widely applicable to a range of other clinical and/or research applications, all relying also on MGRS computation, and therefore requiring multi-SNP genotyping for each patient. Such applications are currently developed in a wide range of diseases, including coronary heart disease [18], liver diseases [19] and acute lymphoblastic leukemia [20]. For these, the methodology reported in this paper is implementable subject to optimization of primers concentration, to building a standardized uniplex pyro-signals-based dictionary and to carrying out multiplex signal analysis with the newly available AdvISER-MH-PYRO function.
